# Development of a Novel Colorimetric pH Biosensor Based on A-Motif Structures for Rapid Food Freshness Monitoring and Spoilage Detection

**DOI:** 10.3390/bios14120605

**Published:** 2024-12-10

**Authors:** Jiajia Wang, Huiyuan Wang, Hongmin Zhang, Shiqi Yang, Keqiang Lai, Donglei Luan, Juan Yan

**Affiliations:** International Research Center for Food and Health, Laboratory of Quality and Safety Risk Assessment for Aquatic Products on Storage and Preservation (Shanghai), Ministry of Agriculture, Shanghai Engineering Research Center of Aquatic-Product Process & Preservation, College of Food Science and Technology, Shanghai Ocean University, Shanghai 201306, China

**Keywords:** A-motif, pH-responsive DNA nanostructures, smart biosensor, colorimetric detection strategy, food safety

## Abstract

Accurate methods for assessing food freshness through colorimetric pH response play a critical role in determining food spoilage and ensuring food quality standards. This study introduces a novel unlabeled DNA sequence, poly-dA_20_, designed to exploit the colorimetric properties of both the single strand and the fold-back A-motif structure in conjunction with gold nanoparticles (AuNPs) under varying pH conditions. When exposed to storage temperatures of 4 °C and 25 °C, the color variations in the AuNP solution, influenced by pH level changes in mutton and sea bass samples’ different storage periods, are easily discernible to the naked eye within a minute. The ratio of UV absorption values at 527 nm and 700 nm (A_527_/A_700_) demonstrates a strong linear correlation with both the storage duration and pH of the food samples. Furthermore, a comprehensive analysis combining the total volatile basic nitrogen (TVB-N) value with the A_527_/A_700_ ratio is employed for precise assessment of food freshness. The innovative pH-responsive sensing strategy not only provides a new approach for on-site food freshness and spoilage detection systems but also serves as a valuable tool for pH-related biological detection in clinical diagnostic applications.

## 1. Introduction

The escalating global concern regarding food safety issues arising from spoilage has become increasingly prominent [1]. Statistics from the Food and Agriculture Organization of the United Nations reveal that over a third of worldwide food waste can be ascribed to spoilage [2,3]. Monitoring food freshness and detecting spoilage have paramount importance in ensuring food safety, elevating food quality, reducing food wastage, and propelling advancements in the food industry. Current approaches to spoilage detection primarily revolve around the identification of biogenic amines [4,5,6], noxious gases [7,8], and microorganisms [9,10,11]. Techniques such as high-performance liquid chromatography (HPLC) [12], capillary electrophoresis [13], Raman spectroscopy [14], liquid chromatography–tandem mass spectrometry [15], and microbial culture [16,17] are commonly employed for detection. However, these methods face challenges due to the complexity and costliness of equipment, intricate detection processes, prolonged detection time, and the necessity for trained personnel to operate them. Furthermore, although portable sensors based on these technologies partially address issues related to cost and equipment, their reliance on specialized instruments and the technical expertise required for detection hinder their application in rapid on-site or household food spoilage monitoring [4,18,19]. Hence, the development of simple, rapid, and portable detection methods, particularly those suitable for on-site testing and potentially for household use, holds significant importance in addressing food safety concerns related to spoilage [18,20].

During the process of food spoilage, the breakdown of proteins by bacteria leads to the production of alkaline compounds, causing an elevation in the food’s pH, while the fermentation of glucose produces acetic acid or organic acids, leading to a decrease in pH [21]. Consequently, the pH value serves as a crucial indicator for evaluating food freshness and the extent of spoilage [22]. Ongoing research predominantly focuses on developing acid–base indicators utilizing natural pigments and synthetic dyes [23]. Natural pigments are eco-friendly, but they exhibit poor stability. In contrast, synthetic dyes are cost-effective, stable, and versatile, albeit with concerns regarding toxicity and carcinogenicity [24]. However, both types still have limitations in practical applications [25], highlighting the urgent necessity for a new material demonstrating stable performance, strong biocompatibility, and sensitivity to pH variations in the domain of food freshness evaluation [26].

Apart from its conventional role as a carrier of genetic information, DNA has emerged as a novel functional material characterized by excellent stability and biocompatibility, with diverse applications in biosensing [27,28]. Various responsive DNA nanostructures, including i-motif, A-motif, and G-quadruplex, undergo structural transformation in response to specific stimuli such as pH, temperature, and ionic strength [29,30]. These structures find extensive utility in biosensors [31], drug delivery systems [32], food safety detection [33], environmental monitoring [34], and various other fields [35]. The A-motif, a DNA sequence comprising exclusively adenine (A), can adopt a double-stranded configuration under acidic conditions and an irregular coiled form under neutral conditions [36,37,38]. While the A-motif’s presence in DNA or RNA plays a pivotal role in gene regulation, protein synthesis, and other biological processes, the utilization of sensing strategies based on the pH responsiveness of the A-motif in food spoilage detection remains relatively limited.

Moreover, colorimetric assays hold significant value in sensors due to their simplicity and cost-effectiveness [39]. Gold nanoparticles (AuNPs) are commonly utilized in these assays owing to their remarkable optical properties, high molar extinction coefficients, and ease of functionalization [40]. These sensors operate by inducing a red shift in the plasmon resonance spectrum of AuNPs through modification in size, medium environment, and distance, resulting in a color change in the solution from red to a clustered blue-gray or purple shade [41]. This spectral shift facilitates quantitative detection through both visual observations and UV–visible absorption spectroscopy [42]. Single-stranded DNA (ssDNA) exhibits a good affinity for the surface of AuNPs by flexibly twisting the sugar–phosphate backbone bond, even in the absence of sulfhydryl modification and the resulting Au-S bond [43]. Notably, sequences rich in adenine, such as poly-dA, have demonstrated significant affinity with the AuNP surface, providing robust resistance to salt-induced aggregation and driving advancements in material biofunctionalization and sensing system construction [44,45,46].

Hence, to shorten the response time of food spoilage sensors, enhance their stability in long-term monitoring of food spoilage, and reduce the operational complexity of the sensors, we have developed a colorimetric pH detecting strategy based on the A-motif for rapid on-site or household detection of food spoilage. The poly-dA_20_, a DNA sequence composed entirely of adenine, undergoes a structural transition from a folded double-stranded configuration (A-motif) under acidic conditions to an irregular single-stranded form under neutral conditions. Unmodified AuNPs exhibit distinctive adsorption characteristics for the two DNA states, allowing for effective differentiation between single-stranded and double-stranded DNA [47]. Through the integration of unmodified AuNPs with pH-responsive A-motif probes, a straightforward, user-friendly, rapid, sensitive, and real-time colorimetric pH response sensing system has been engineered for evaluating both food freshness and spoilage.

## 2. Materials and Methods

### 2.1. Materials

Chloroauric acid hydrate (HAuCl_4_, 99%, Shanghai Titan Technology Co., Ltd., Shanghai, China), trisodium citrate (Na_3_C_6_H_5_O_7_·2H_2_O), sodium dihydrogen phosphate (NaH_2_PO_4_), and disodium hydrogen phosphate (Na_2_HPO_4_) were sourced from China National Pharmaceutical Group Corporation Chemical Reagent Co., Ltd., Shanghai, China. All chemical reagents were of analytical grade quality. Ultrapure water was obtained using the Milli-Q system (Barnstead, Dubuque, TX, USA). The DNA sequences employed in the research were procured from Huzhou HeMa Biological Technology Co., Ltd., Huzhou, China, and underwent purification via HPLC. All synthetic DNA sequences were tabulated in Table 1.

### 2.2. Apparatus

The apparatus included a UV–vis spectrophotometer (Shanghai Lengguang Technology Co., Ltd., Shanghai, China), circular dichroism spectrometer (Applied Photophysics Ltd., Leatherhead, UK), Hitachi F-7100 fluorescence spectrophotometer (Hitachi, Tokyo, Japan), magnetic heating stirrer (Jiangsu Kexi Instrument Co., Ltd., Changzhou, China), pH meter (Mettler Toledo Technology (China) Co., Ltd., Shanghai, China), Mixer 4K miniature vortex mixer (Sangon Biotech (Shanghai) Co., Ltd., Shanghai, China), HCM100-Pro thermostatic oscillating metal bath (Dalong Xingchuang Experimental Instrument (Beijing) Co., Ltd., Beijing, China), FSH-2A adjustable high-speed homogenizer, and Eppendorf Centrifuge 5417R small benchtop refrigerated centrifuge.

### 2.3. Characterization of A-Motif Fold-Back Structure

First, 1 μL of 10 μM poly-dA_20_ chains were dissolved in aqueous solutions at pH levels of 4, 7, and 8, each at a final concentration of 100 nM, and incubated at 37 °C for 3 min. Subsequently, the absorption spectra of the DNA chains were recorded using a circular dichroism spectrometer spanning the wavelength range of 200 to 300 nm. Additionally, the fluorescence spectra of Cy3-poly-dA_20_ were obtained between 550 and 750 nm using a fluorescence spectrophotometer under the same pH and temperature conditions. The fluorescence spectroscopy of the Cy3-poly-dA_20_-BHQ2 probe followed a similar experimental approach.

### 2.4. Processing Methods of Real Food Samples

The fresh mutton meat (200 g) and sea bass minced meat (200 g) were stored separately in polyethylene bags at temperatures of 4 °C and 25 °C. The 4 °C samples were collected once daily for a total of seven days. The 25 °C samples were collected at 0, 8, 16, 24, 48, and 72 h, with a total sampling period of 72 h. To prepare the samples, 8 g of each mince was homogenized in 20 mL of deionized water (12,000 rpm/minute, 25 °C, 2 min) and then subjected to centrifugation (12,000 rpm/minute, 4 °C, 15 min) to obtain the supernatant. Following the pH measurement of the supernatant, 1 μL of 10 μM poly-dA_20_ was added to 99 μL of supernatant to achieve a final concentration of 100 nM, and the mixture was homogenized for 3 min at 37 °C. Then, 10 μL of this solution was combined with 100 μL of a 5 nM AuNP (∼30 nm) solution and vortexed for 3 min at 25 °C. Finally, 19.4 μL of 1 M NaCl was added to reach a final NaCl concentration of 0.15 M. After thorough mixing at room temperature, the color of the solution was observed, and the UV–vis absorption spectra of the solution were recorded.

### 2.5. Determination of Total Volatile Basic Nitrogen (TVB-N)

The determination of TVB-N value was referenced to the work of Huang et al. [48].

## 3. Results

### 3.1. Design Principles for Developing pH Colorimetric Sensing Strategy Utilizing the A-Motif

**Figure 1:**
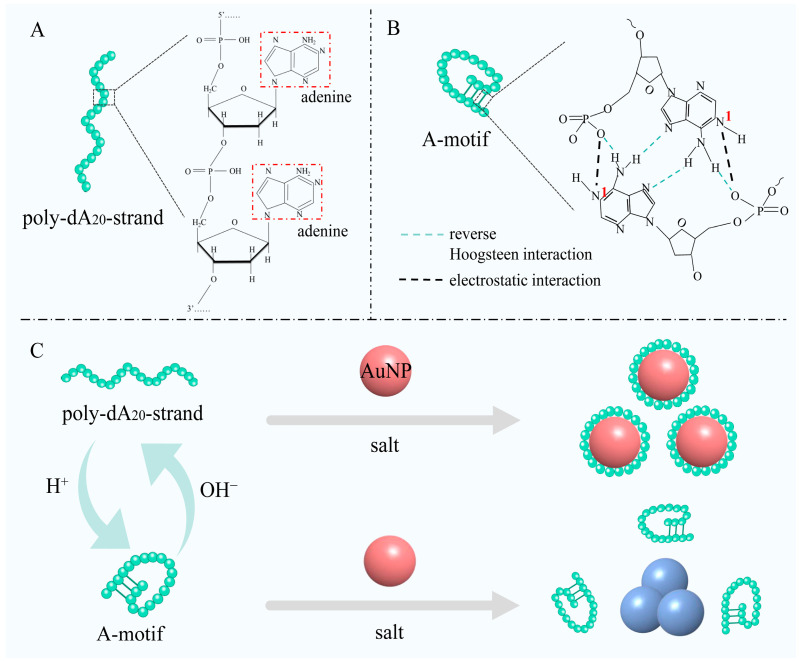
(**A**) Schematic diagram of a short oligonucleotide sequence composed of homopolymeric deoxyadenosines, where structural units consisting of phosphate groups, deoxyribose, and adenine are linked via phosphodiester bonds. (**B**) shown the folding structure of A-motif caused by the base pairing scheme in AH^+^ -H^+^A base pairs comprising protonated adenosines. Red 1 represents the N1 protonated site on adenines. (**C**) Schematic diagram of colorimetric biosensing strategy based on A-motif structure.

The study utilized an oligonucleotide sequence consisting of a 20-homopolymeric deoxyadenosine (poly-dA_20_) strand connected by phosphodiester bonds, prior to structural changes. This strand features a 5′ end terminating with a phosphate group and a 3′ end with a hydroxyl group. In a neutral pH environment, the strand adopts a random coil conformation. However, under acidic conditions, it undergoes a structural transformation into an A-motif, characterized by the formation of regions with parallel double-stranded (ds) structures facilitated by reverse Hoogsteen base pairing and electrostatic interactions [36]. The schematic diagram visually illustrates the structural variances between the poly-dA_20_ strand and the A-motif structure (A and B, Figure 1).

The structural transformation of poly-dA_20_ chains in varying pH conditions, along with the differential interactions of unmodified gold nanoparticles (AuNPs) with ssDNA and dsDNA in high-salt environments, has led to the development of a colorimetric method based on the A-motif for detecting food spoilage and monitoring food freshness (C, Figure 1). In a neutral pH setting, poly-dA_20_ chains exist as liner single strands, interacting collectively with the AuNP surface, causing hydrophobic collapse and enhancing adsorption affinity. In saline conditions, the poly-dA_20_ sequences shield AuNPs from salt-induced aggregation, preserving the red color of the solution [44,49]. In contrast, when exposed to an acidic environment, the poly-dA_20_ strand transitions into the A-motif, diminishing its affinity for AuNPs and leading to the aggregation of AuNPs in high-salt solution, resulting in hues of purple or blue-gray. These observable color changes, along with alternations in the UV absorption spectrum of the AuNPs that can be analyzed using a UV–visible spectrometer, facilitate both the qualitative and quantitative assessment of food sample freshness.

### 3.2. Structural Characterization of A-Motif Under pH Changes

**Figure 2:**
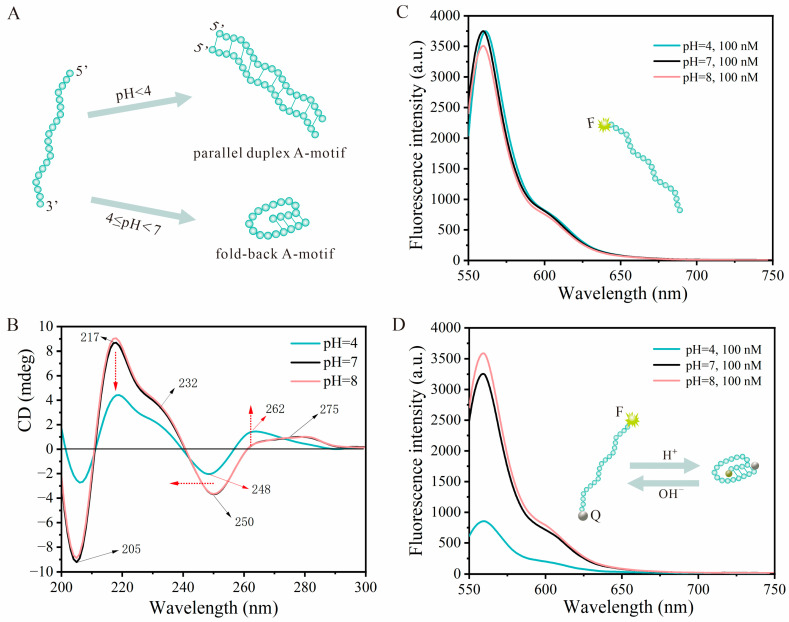
(**A**) Schematic diagram of the transformation of the poly-dA_20_ strand from a random coil state to two distinct A-motif structures at varying pH conditions (pH < 4 and 4 ≤ pH < 7). (**B**) Circular dichroism results of the poly-dA_20_ probes under different pH conditions. The red dotted arrows represent the trend of the CD spectrum as pH decreases from 7 to 4. (**C**) Fluorescence spectra of Cy3-DNA probes at a concentration of 100 nM under varying pH conditions (pH = 4, 7, and 8). The insert diagram illustrates the structure of the Cy3-poly-dA_20_ strand. (**D**) Fluorescence spectra of Cy3-A_20_ probes labeled with quenched (Q) groups, BHQ2, at a concentration of 100 nM in different pH environments (pH = 4, 7, and 8). The insert diagram illustrates the structural transformation of the F-A_20_-Q probes in response to acidic and alkaline environments.

Previous studies have shown that poly-dA_20_ strands, when present at high concentrations in environments with a pH < 4, tend to adopt a parallel double-stranded A-motif structure [50]. This is attributed to the higher degree of protonation in low-pH settings, which facilitates the formation of parallel double-stranded structures through intermolecular AH^+^-H^+^A base pairs. However, within the pH range of 4 to just below 7, the low molar ratio of protons to poly-dA_20_ allows the probe to maintain a fold-back A-motif structure under these conditions (Figure 2A). Whether forming parallel duplex A-motifs or fold-back A-motifs, the presence of a double-chain structure enables the combination of surface plasmon resonance (SPR) properties of AuNPs for the development of colorimetric sensing strategies. As practical application under pH < 4 conditions is limited, particularly in the context of food sample analysis, the study focused on investigating the structure and sensing performances of the A-motif in the pH range 4~8.

The ability of the poly-dA_20_ strand to undergo structural transformation with variation in pH from a linear single-chain state to a fold-back double-strand structure forms the foundation of this sensing strategy. To validate this property, we initially employed circular dichroism (CD) and fluorescence spectrometry methods. Ultraviolet–CD spectroscopy in the UV region serves as a valuable tool for distinguishing between various DNA structures [51]. In the CD spectra shown in Figure 2B, at pH 7 (black line), poly-dA_20_ showed a characteristic CD trace with a strong positive maximum at 217 nm with a shoulder at 232 nm, a weak positive band at 275 nm, and negative bands centered at 250 nm and 205 nm, indicative of a single-stranded poly-dA_20_ [52]. In contrast, the CD spectrum of poly-dA_20_ at pH 4 (blue line) displayed distinct features, including a decline in the intensity of the positive band at 217 nm and the emergency of a strong positive peak at 262 nm. These findings align with the previous literature on poly-dA_20_ at pH 4 [53]. Moreover, our observation at pH 8 showed minimal variation in the CD spectrum compared to pH 7, indicating that the poly-dA_20_ probe maintained a single-stranded state even with the pH increase from 7 to 8. These results confirm the excellent response of the poly-dA_20_ strand to pH variations, demonstrating two distinct state transitions between a linear chain and a fold-back structure.

In addition to CD analysis, fluorescence spectroscopy was also utilized to assess how pH variations affect the structures of DNA probes. In Figure 2C, Cy3-poly-dA_20_ demonstrated robust fluorescence at pH 7 and 8, with the fluorescence intensity remaining unaffected even when the pH dropped to 4. This suggests that pH variations do not compromise the fluorescence intensity of Cy3 attached to poly-dA_20_ strands. The fluorescence intensity of the Cy3-poly-dA_20_-BHQ2 probe was analyzed to track the transition from a single-stranded state to a fold-back A-motif structure. As depicted in Figure 2D, the fluorescence of the probe significantly increased as the solution pH ranged from 4 to 7 (blue and blank lines). The findings indicate that with pH elevation, a decrease in FRET efficiency from Cy3 to BHQ2 occurred, likely due to the greater distance between BHQ2 and Cy3. Furthermore, the results indicate that a pH of 4 triggered the formation of a fold-back structure, confirming the successful conversion from a single strand to a fold-back double strand structure as the pH changed from 4 to 7. Moreover, at pH 8, the fluorescence value slightly increased, suggesting that the Cy3-poly-dA_20_-BHQ2 probe remained in an unfold single-chain state. These observations are consistent with the results of CD characterization, offering insights into the feasibility of designing pH-responsive biosensors utilizing the A-motif structure.

### 3.3. Structural Characterization of A-Motif Under pH Change

**Figure 3:**
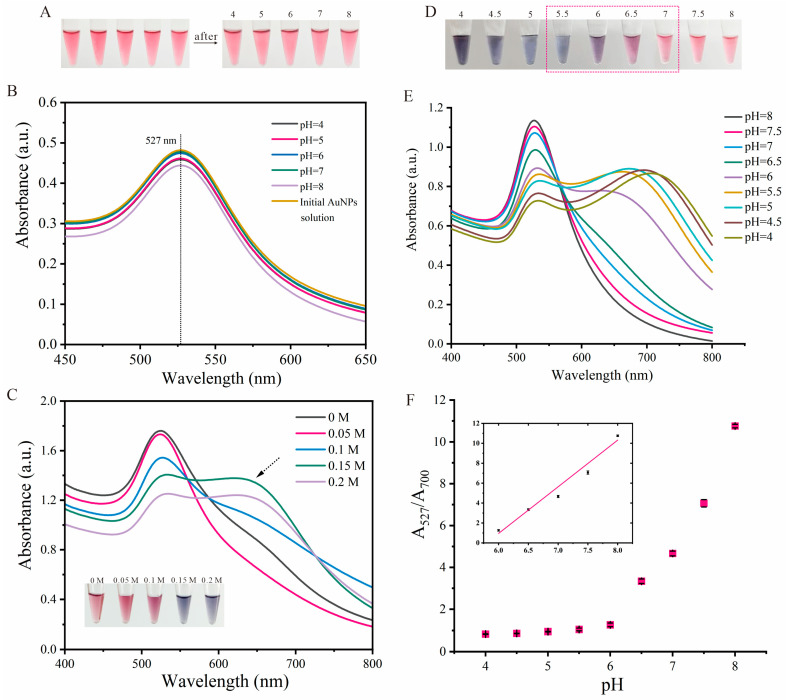
(**A**) Visual images before and after the introduction of poly-dA_20_ chains into AuNPs solutions under varying pH conditions. (**B**) UV–vis absorption spectra of poly-dA_20_/AuNPs mixed solutions at different pH levels. (**C**) UV–vis absorption spectra of AuNPs at various final concentrations of NaCl. Insert: Visual images of AuNPs at various final NaCl concentrations. (**D**) Visual color variations in poly-dA_20_/AuNPs system at different pH values after introducing a final concentration of 0.15 M NaCl. (**E**) UV–vis absorption spectra of poly-dA_20_/AuNPs system at different pH values after introducing a final concentration of 0.15 M NaCl. (**F**) Plot of the absorption ratio (A_527_/A_700_) at different pH values. Insert: a locally linear relationship between absorbance ratio and pH value from pH 6.0 to 8.0. The error bars represent the standard deviation of three measurements.

Prior to developing a pH colorimetric sensor based on the A-motif structure, a solution of 30 nm AuNPs solution (100 µL) with a concentration of 5 nM was mixed with poly-dA_20_ chains at a final concentration of 100 nM. These poly-dA_20_ chains were dissolved in aqueous solutions at varying pH levels (4, 5, 6, 7, 8). Following thorough mixing at room temperature, it was observed that the color of the AuNPs solution remained largely unchanged (Figure 3A). Additionally, minimal deviations were noted in the UV–vis absorption spectra compared to the initial AuNP solution (Figure 3B), indicating that the poly-dA_20_ at different pH values did not induce aggregation of the AuNPs. The primary factor influencing the colorimetric response of the AuNPs is the disturbance of the stable electric double-layer structure on the nanoparticle’s surface by electrolytes present in the salt solution, which leads to color changes due to aggregation. Adjustment of the salt concentration allows for adjustments to the surface characteristics of the colloidal AuNPs to enhance the sensor’s sensitivity and detection capabilities. In simulated experiments, various concentrations of NaCl solution were introduced to achieve final concentration of 0, 0.05, 0.1, 0.15, and 0.2 M to assess the salt tolerance of the bare AuNPs. The results indicate a significant alteration in the color of AuNPs when the final salt concentration reached 0.15 M (Insert, Figure 3C). Concurrently, a new broad peak emerged in the UV–vis absorption spectra within the range of 550~700 nm (Figure 3C), supplementing the distinctive absorption peak at 527 nm for the 30 nm AuNPs. Therefore, 0.15 M was identified as the threshold NaCl concentration required to trigger the aggregation of AuNPs, establishing it as the optimal electrolyte condition for subsequent experiments.

Subsequently, poly-dA_20_ chains dissolved in solutions with varying pH levels (ranging from 4 to 8 in 0.5 pH unit increments) were introduced to the AuNP solutions, followed by the addition of NaCl to reach a final concentration of 0.15 M. Notably, at pH 4, the solutions rapidly shifted from red to blue, while at pH 8, the solution maintained its red color (Figure 3D). Particularly, the most pronounced color transformation of AuNPs was an observed trend within the pH range of 5.5 to 7 (red dashed box, Figure 3D). The transition towards blue in the AuNPs as the pH decreased indicated an increase in the nanoparticles’ aggregation state. These observations suggest that while the oligonucleotide chain containing adenine exhibits excellent adsorption properties to the surface of AuNPs, it loses its ability to adhere effectively to the AuNPs surface as it transitions from a single chain to a fold-back structure. This transformation was corroborated by the outcomes of the UV–vis absorption spectra analysis (Figure 3E). Specifically, at the pH values greater than 7, the poly-dA_20_ chains were in a fully extended single-stranded configuration capable of adsorbing onto the AuNPs’ surface, preserving good dispersibility in the presence of salt and displaying a red color with a singular absorption peak at 527 nm. However, within the pH range of 4 to 7, the poly-dA_20_ chains adopted a fold-back A-motif structure, leading to aggregation and a color shift to blue, accompanied by the emergence of a new broad absorption peak. The correlation between the alteration in solution pH and the shift in absorbance prompted the utilization of the absorbance ratio of wavelengths 527 nm and 700 nm (A_527_/A_700_) as a method to measure and quantify the color change of the AuNPs. Furthermore, the impact of pH on the assembly state of AuNPs is illustrated in Figure 3F. A sharp shift in the absorption ratio was observed at pH 6.0, suggesting a substantial conformational change in the DNA chains around this pH, specifically the unfolding of the A-motif structure. Additionally, the data points within the pH range of 6 to 8 exhibited a high level of linearity (Insert, Figure 3F), demonstrating the potential applicability of this approach in biosensing detection.

### 3.4. Determination of Actual Food Sample Freshness with A-Motif-Based Colorimetric pH Biosensor

**Figure 4:**
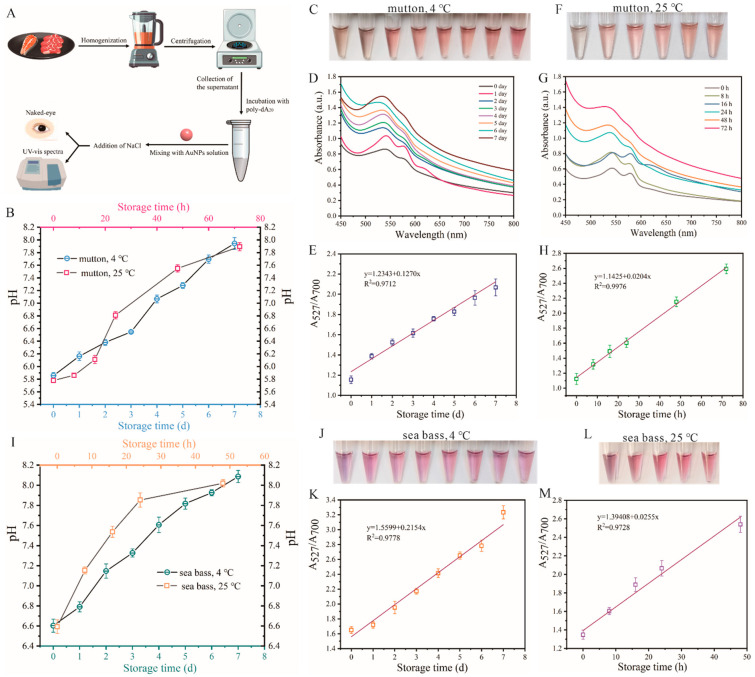
(**A**) Schematic diagram of sample handling process for mutton and sea bass. (**B**) The time–pH curve of mutton during storage at 4 °C and 25 °C. (**C**) Visual images of AuNP colorimetric pH sensor based on A-motif to mutton samples stored at 4 °C for different days. The images depict the progression of 0, 1, 2, 3, 4, 5, 6, and 7 days from left to right. (**D**) UV–vis absorption spectra of mutton samples stored for different days at 4 °C using A-motif colorimetric pH sensing system. (**E**) The linear correlation between the storage time of mutton samples at 4 °C and the A_527_/A_700_ ratio using the colorimetric sensing system. (**F**) Visual images of AuNPs colorimetric pH sensor based on A-motif to mutton samples stored at 25 °C for different hours. The images depict the progression of 0, 8, 16, 24, 48, and 72 h from left to right. (**G**) UV–vis absorption spectra of mutton samples stored for different hours at 25 °C using A-motif colorimetric pH sensing system. (**H**) The linear correlation between the storage time of mutton samples at 25 °C and the A_527_/A_700_ ratio using the colorimetric sensing system. (**I**) The time–pH curve of sea bass during storage at 4 °C and 25 °C. (**J**) Visual images of AuNP colorimetric pH sensor based on A-motif to sea bass samples stored at 4 °C for different days. The images depict the progression of 0, 1, 2, 3, 4, 5, 6, and 7 days from left to right. (**K**) The linear correlation between the storage time of sea bass samples at 4 °C and the A_527_/A_700_ ratio using the colorimetric sensing system. (**L**) Visual images of AuNPs colorimetric pH sensor based on A-motif to sea bass samples stored at 25 °C for different hours. The images depict the progression of 0, 8, 16, 24, and 48 h from left to right. (**M**) The linear correlation between the storage time of sea bass samples at 25 °C and the A_527_/A_700_ ratio using the colorimetric sensing system.

To assess the performance of the sensor in monitoring the freshness of actual food samples, we utilized the colorimetric pH biosensing method to track the pH levels of mutton and sea bass samples across varying temperature and time settings. In practical applications, the complexity of food samples, such as variability in sample preparation and the sample’s inherent color, can interfere with colorimetric sensor performance. To minimize these factors, we used a standardized preparation method, including homogenization and centrifugation followed by analysis of the supernatant. As depicted in Figure 4A, the processed mutton and sea bass samples underwent several steps: homogenization, centrifugation, exposure to poly-dA_20_ chains, mixing with AuNPs solution, and addition of NaCl solution. Visual monitoring of color variations in the solutions and analysis of UV–vis spectra were conducted. Figure 4B illustrated the pH trend of mutton samples over time at storage temperatures of 4 °C and 25 °C. The gradual increase in pH over time highlights its significance as a crucial indicator of food freshness. As the storage temperature transitioned from 4 °C to 25 °C, the pH of the samples rose from approximately 5.8 to around 8, with the duration of this transition decreasing from 7 days to 72 h. Moreover, the color evolution of mutton samples, as depicted in Figure 4C, showed that fresh mutton samples (0 days) exhibited gray AuNPs, indicating a certain degree of aggregation. With prolonged storage, the pH value increased, the dispersion of AuNPs improved, causing a color shift from gray to red. Particularly in the initial stages of food storage, the pH value typically fluctuates within the range of 5.5 to 7.0, resulting in noticeable color variations. These color variations were further supported by the UV–vis absorption spectra in Figure 4D. A strong linear relationship was observed between the storage time of mutton samples and the absorbance ratio of AuNPs at two distinct wavelengths (A_527_/A_700_) in a 4 °C storage setting (Figure S1A) (y = 1.2343 + 0.1270x, R^2^ = 0.9712), as shown in Figure 4E. Similarly, at 25 °C, Figure 4F showcases visual changes in mutton samples over storage time based on the A-motif structure of the AuNPs colorimetric pH sensor, allowing for naked-eye observation of color changes. As the storage duration progresses, the increase in pH leads to a color change in AuNPs from gray to red. The UV–vis spectra analysis in Figure 4G confirms a robust linear correlation between the preservation time of mutton samples at 25 °C and the A_527_/A_700_ ratio (Figure S1B) (y = 1.1425 + 0.0204x, R^2^ = 0.9976), as illustrated in Figure 4H. Compared to previous studies, this sensor demonstrates a faster response time [54,55]. Within the pH range of 5.8 ± 0.1 units to 7.9 ± 0.1 units, it exhibits a rapid response to pH changes, with a response time of only 3 min. Furthermore, the standardized pre-treatment process of food samples and the specific response of A-motif to pH significantly reduce the impact of complex matrix components in food on the system, thereby enhancing the sensitivity and repeatability of the method.

To validate the extensive applicability of the colorimetric pH sensing system, sea bass samples were also examined in the study. Both fish samples stored at 4 °C and 25 °C underwent comprehensive testing and analysis to observe pH trends over time. Notably, the pH of fish samples exhibited a more pronounced increase at 25 °C compared to mutton samples, reaching a pH of 8 within 48 h. Therefore, our analysis focused on data collected within the initial 48 h to capture the changes in fish samples under the 25 °C storage condition. The color changes of AuNPs from purple to red in these samples under varying storage conditions were closely monitored (Figure 4J,L), along with the UV–vis absorption spectra (Figure S2A,B). Moreover, the correlation between storage duration and the A_527_/A_700_ ratio was also investigated (Figure S2C,D). The results also reveal a linear equation describing the relationship between storage duration and A_527_/A_700_ for sea bass samples stored at 4 °C (y = 1.5599 + 0.2154x, R^2^ = 0.9778, Figure 4K) and 25 °C (y = 1.3941 + 0.0255x, R^2^ = 0.9728, Figure 4M). These outcomes highlight the successful application of the pH sensing methodology, which relies on the poly-dA_20_ chain and A-motif structure transformation, for rapid assessment of food freshness and accurate quantitative evaluation of food pH levels.

### 3.5. Monitoring Food Samples Freshness by Applying pH Sensing System and TVB-N Index

**Figure 5:**
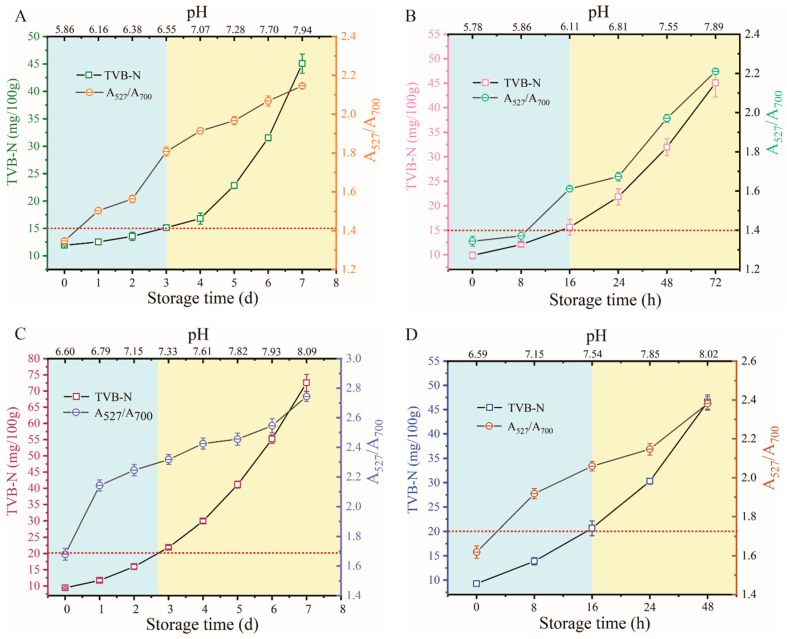
(**A**) Variations in TVB-N values and A_527_/A_700_ ratios in mutton samples over preservation time and different pH values during storage at 4 °C. (**B**) Variations in TVB-N values and A_527_/A_700_ ratios in mutton samples over preservation time and different pH values during storage at 25 °C. (**C**) Variations in TVB-N values and A_527_/A_700_ ratios in sea bass samples over preservation time and different pH values during storage at 4 °C. (**D**) Variations in TVB-N values and A_527_/A_700_ ratios in sea bass samples over preservation time and different pH values during storage at 25 °C. The red dashed lines correspond to the threshold TVB-N values, set at 15 mg/100 g for mutton samples and 20 mg/100 g for sea bass samples. The blue area signifies the fresh stage of the food sample, while the yellow area indicates the spoilage stage of the food sample.

The research method was further compared to the conventional food freshness assessment method based on the TVB-N index. As meat undergoes enzymatic and bacterial processes during storage, protein and amino acids break down into nitrogenous compounds like ammonia and amines, reflected in the TVB-N levels [48]. The accumulation of TVB-N with prolonged storage time serves as a crucial indicator of protein degradation, playing a significant role in determining changes in food freshness and nutritional value deterioration. According to the Chinese standard GB 5009.228-2016, TVB-N values exceeding 15 mg/100 g for mutton and 20 mg/100 g for sea bass signify the onset of spoilage. Therefore, thresholds of 15 mg/100 g for mutton and 20 mg/100 g for sea bass were adopted for freshness evaluation, represented by the red dashed lines in Figure 5. As depicted in Figure 5A,B, the TVB-N values of both mutton sample sets (stored at 4 °C and 25 °C) exhibited varying degrees of increase over time. After 3 days at 4 °C and 16 h at 25 °C, the TVB-N levels of the mutton samples surpassed the threshold, corresponding to an A_527_/A_700_ ratio of approximately 1.4, with pH readings of 6.55 and 6.11 for the respective groups. Similarly, sea bass samples stored at 4 °C reached a TVB-N value of 20 mg/100 g after 2 to 3 days, with an associated A_527_/A_700_ value of 1.7 and pH reading of 7.1 ± 0.1 units (Figure 5C). At 25 °C, the TVB-N value exceeded the threshold within 16 h, yielding an A_527_/A_700_ value of about 1.7 and a pH of 7.54 (Figure 5D). By integrating the colorimetric strategy with the TVB-N method, a precise assessment of food freshness was achieved. It is important to note that the diversity in pH variation levels among various food samples makes it a challenge to establish a universal criterion for assessing the freshness and spoilage of food using the current approach. The methodology is tailored to individual food items, enabling precise monitoring of their freshness levels. Through the amalgamation of diverse analytical techniques and the thresholds, a comprehensive and accurate assessment of food quality can be achieved.

## 4. Conclusions

In conclusion, the study successfully developed a colorimetric pH biosensor using AuNPs and A-motif structure, showing promising applications in detecting the freshness of new products. The visual imaging results indicate that the color and the UV absorption characteristics of the AuNPs were highly responsive to variations in food pH levels under storage conditions of 4 °C and 25 °C. Particularly notable was the significant color change observed when the pH fell within the range of 5.5~7.0, offering a clear indicator of changes in food freshness, especially during the early stages of storage. Furthermore, by integrating the colorimetric pH sensing method with the TVB-N index, the pH trends associated with changes in food freshness were determined against the backdrop of the TVB-N freshness threshold. These complementary methods were mutually referenced to enable precise monitoring of food freshness. In comparison to other approaches for monitoring food spoilage or freshness, the A-motif-based colorimetric pH sensor excelled in terms of its small sample requirements, ease of operation, and rapid and sensitive detection capabilities, as well as its intuitive and visual monitoring approach, catering to the demands of both stores and households for quick on-site assessment of food freshness. The outcomes of this study are poised to advance the field of smart sensors for pH-responsive applications in food safety detection and packaging, with prospects for fine-tuning the pH application range through the integration of other pH-responsive DNA nanostructures like the i-motif. Leveraging the correlation between the digital signals from colorimetric visual images and food freshness indices, a smartphone auxiliary system will be developed to facilitate real-time, non-destructive, and intelligent monitoring of food freshness.

## Figures and Tables

**Table 1:** DNA probe sequences required in this work.

DNA	Sequence (5′~3′)
poly-dA_20_	AAAAAAAAAAAAAAAAAAAA
Cy3-poly-dA_20_	Cy3-AAAAAAAAAAAAAAAAAAAA
Cy3-poly-dA_20_-BHQ2	Cy3-AAAAAAAAAAAAAAAAAAAA-BHQ2

## Data Availability

The original contributions presented in the study are included in the article and Supplementary Material, further inquiries can be directed to the corresponding authors.

## Supplementary Materials

The following supporting information can be downloaded at https://www.mdpi.com/article/10.3390/bios14120605/s1: Preparation of gold nanoparticles (AuNPs); Figure S1: A_527_/A_700_ values of mutton samples at 4 °C and 25 °C over various storage durations; Figure S2: A_527_/A_700_ values of sea bass samples at 4 °C and 25 °C over various storage durations [56].
