# Supplementary material for: Development of a Novel Colorimetric pH Biosensor Based on A-Motif Structures for Rapid Food Freshness Monitoring and Spoilage Detection
Source: Biosensors (Basel). 2024 Dec 10;14(12):605. doi: 10.3390/bios14120605 (PMC11674138; doi:10.3390/bios14120605)
Supplement: Supplementary file 1 [file biosensors-14-00605-s001.zip › biosensors-3313618-supplementary.pdf]

biosensors

Supporting Information

Development of a Novel Colorimetric pH Biosensor Based on A-Motif Structures for Rapid Food Freshness Monitoring and Spoilage Detection

Jiajia Wang †, Huiyuan Wang †, Hongmin Zhang, Shiqi Yang, Keqiang Lai, Donglei Luan * and Juan Yan *

International Research Center for Food and Health; Laboratory of Quality and Safety Risk Assessment for Aquatic Products on Storage and Preservation (Shanghai), Ministry of Agriculture; Shanghai Engineering Research Center of Aquatic-Product Process & Preservation; College of Food Science and Technology, Shanghai Ocean University, Shanghai 201306, China;m210300892@st.shou.edu.cn (J.W.); d220300089@shou.edu.cn (H.W.); hmzhang@shou.edu.cn (H.Z.); m230301011@st.shou.edu.cn (S.Y.); kqlai@shou.edu.cn (K.L.)

† These authors contributed equally to this work.

* Correspondence: dlluan@shou.edu.cn (D.L.); j-yan@shou.edu.cn (J.Y.)

Citation: Wang, J.; Wang, H.; Zhang, H.; Yang, S.; Lai, K.; Luan, D.; Yan, J. Development of a Novel Colorimetric pH Biosensor Based on A-Motif Structures for Rapid Food Freshness Monitoring and Spoilage Detection. *Biosensors* **2024**, *14*, x. https://doi.org/10.3390/xxxxx

Received: 29 October 2024
Revised: 30 November 2024
Accepted: 07 December 2024
Published: date

Copyright: © 2024 by the authors. Submitted for possible open access publication under the terms and conditions of the Creative Commons Attribution (CC BY) license (https://creativecommons.org/licenses/by/4.0/).

1. Preparation of gold nanoparticles (AuNPs)

AuNPs were synthesized using the traditional sodium citrate reduction method outlined by Frens in 1973 [1]. The procedure involved starting with a 0.01% chlorauric acid solution of 100 mL, which was heated to boiling. Subsequently, 0.7 mL of a 1% trisodium citrate solution was rapidly added to the boiling mixture under vigorous agitation. The initially golden chlorauric acid solution transitioned to a purple hue within 2 minutes. The mixture was then boiled for 15 minutes before the heating was discontinued, and stirring was maintained overnight. Lastly, the obtained 30 nm AuNPs solution was filtered through a 0.22 μm aperture filter membrane and stored at 4 °C.

2. Figures

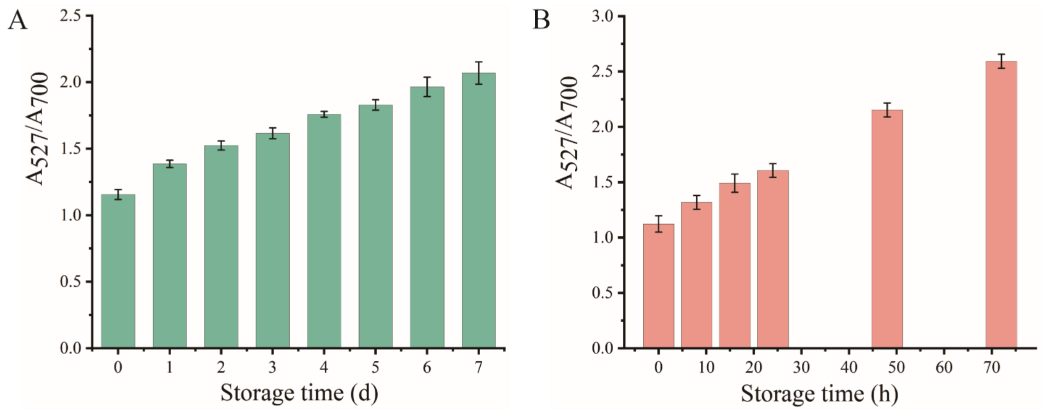

Figure S1. (A) A_{527}/A_{700} ratio values for mutton samples stored at 4 °C over varying durations. (B) A_{527}/A_{700} ratio values for mutton samples stored at 25 °C over different time periods.

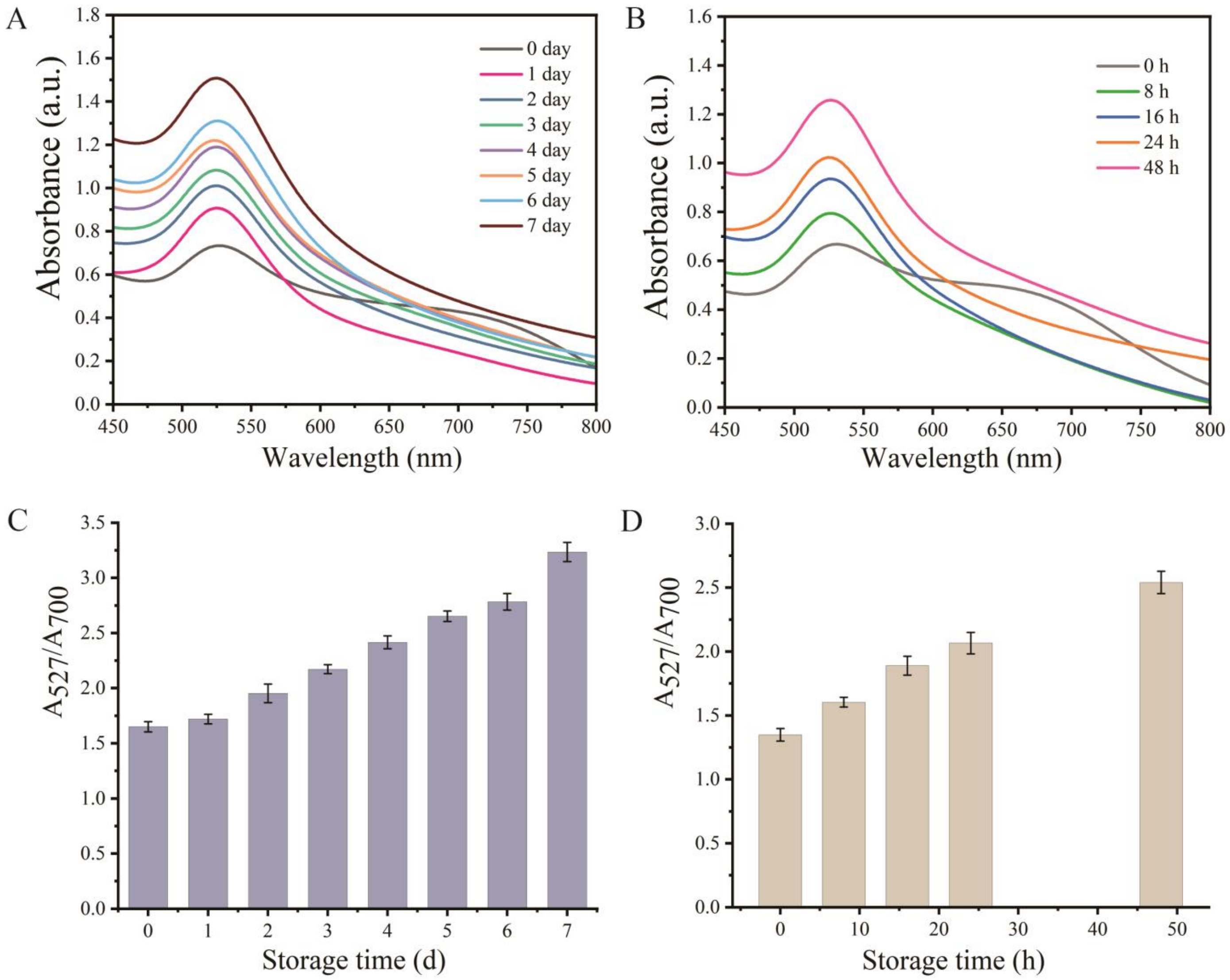

Figure S2 UV-vis absorption spectra for sea bass samples stored at 4 °C (A) and 25 °C (B) for different periods of time. A_{527}/A_{700} ratio values for sea bass samples stored at 4 °C (C) and 25 °C (D) over different time periods.
